# Sulforaphane Ameliorates Bladder Dysfunction through Activation of the Nrf2-ARE Pathway in a Rat Model of Partial Bladder Outlet Obstruction

**DOI:** 10.1155/2016/7598294

**Published:** 2016-06-28

**Authors:** Chong Liu, Huan Xu, Shi Fu, Yanbo Chen, Qi Chen, Zhikang Cai, Juan Zhou, Zhong Wang

**Affiliations:** Department of Urology, Shanghai Ninth People's Hospital, Shanghai Jiao Tong University School of Medicine, Shanghai 200011, China

## Abstract

*Purpose*. We evaluated the effect of sulforaphane (SFN) treatment on the function and changes of expression of Nrf2-ARE pathway in the bladder of rats with bladder outlet obstruction (BOO).* Materials and Methods*. A total of 18 male Sprague-Dawley rats at age of 8 weeks were divided into 3 groups (6 of each): the sham operated group, the BOO group, and the BOO+SFN group. We examined histological alterations and the changes of oxidative stress markers and the protein expression of the Nrf2-ARE pathway.* Results*. We found that SFN treatment could prolong micturition interval and increase bladder capacity and bladder compliance. However, the peak voiding pressure was lower than BOO group. SFN treatment can ameliorate the increase of collagen fibers induced by obstruction. SFN treatment also increased the activity of SOD, GSH-Px, and CAT compared to the other groups. The level of bladder cell apoptosis was decreased in BOO rats with SFN treatment. Moreover, SFN could reduce the ratio of Bax/Bcl-2 expression. Furthermore, SFN could activate the Nrf2 expression with elevation of its target antioxidant proteins.* Conclusions*. The sulforaphane-mediated decrease of oxidative stress and activation of the Nrf2-ARE pathway may ameliorate bladder dysfunction caused by bladder outlet obstruction.

## 1. Introduction

Benign Prostatic Hyperplasia (BPH) is a common proliferative disease in older men, and more than 30% of males over 60 years of age have some degree of bladder outlet obstruction (BOO) due to BPH [1]. BPH clinically presents with lower urinary tract symptoms (LUTS), such as urinary frequency, urgency, nocturia, and urge incontinence, which usually result in urinary tract infections and renal impairment. BOO could induce significant structural and functional changes of the bladder in both laboratorial and clinical researches [2]. However, the molecular mechanisms of how BOO leads to bladder dysfunction remain unclear.

Increasing evidence has shown that cyclic ischemia/reperfusion are major etiologic factors in the progression of bladder dysfunction induced by BOO [3]. Ischemia/reperfusion injury in the bladder could induce the generation of reactive oxygen species (ROS), such as superoxide anions and hydroxyl radicals, and the resultant cellular and subcellular membrane peroxidation [4]. Since ROS is a pathogenic factor in the dysfunctional bladder, antioxidants may be effective for treating bladder dysfunction secondary to BOO. The administration of antioxidants has been shown beneficial for ameliorating bladder dysfunction in animal obstruction models [5, 6]. However, the exact mechanism has not been clarified. Nuclear erythroid related factor 2 (Nrf2) is a transcription factor involved in regulating the cellular antioxidative responses and redox status by promoting the expression of antioxidative genes through the antioxidant response element (ARE) [7, 8]. Sulforaphane (SFN) is a naturally occurring isothiocyanate that is present in cruciferous vegetables such as broccoli. As a chemopreventive compound, SFN has been studied for its antioxidative and inflammatory properties. Its antioxidative ability is mediated by Nrf2, which is bound to the Kelch-like ECH-associated protein 1 (Keap1) in the cytoplasm under basal conditions. SFN promotes Nrf2's translocation into the nucleus and then binding to ARE to induce expression of cytoprotective genes such as heme oxygenase-1 (HO-1), NAD(P)H: quinone oxidoreductase 1 (NQO1). Such genes, in turn, play a major role in the detoxification of ROS produced during ischemia/reperfusion [9, 10].

Previous studies have indicated that the activation of Nrf2 pathway by SFN shows effective protection in various diseases [10–13]. However, whether SFN could protect bladder tissue by activating the Nrf2 pathway in the BOO model has not been well defined.

In the study, we sought to clarify SFN's role in ameliorating bladder dysfunction through a BOO rat model. In addition, whether the Nrf2-ARE pathway was involved in SFN's protection of bladder function was also investigated.

## 2. Materials and Methods

### 2.1. Reagents and Antibodies

Sulforaphane was provided by Cayman Chemical (USA). Anti-Nrf2, HO-1, and NQO1 antibodies were obtained from Abcam (Cambridge, UK); anti-bcl-2, Bax, PCNA, and GAPDH antibodies were acquired from CST (Danvers, MA, USA); goat-anti-rabbit IgG-HRP, goat-anti-mouse IgG-HRP, and DAB detection Kit were obtained from DAKO. The TdT-mediated dUTP Nick-End Labeling (TUNEL) Apoptosis Assay Kit was provided by KeyGEN BioTECH (Nanjing, China). The BCA assay kit was provided by Thermo Scientific. The nuclear protein extraction kit was provided by Beyotime Institute of Biotechnology (Beijing, China). The malondialdehyde (MDA) assay kit, total superoxide dismutase (SOD) assay kit, glutathione peroxidase (GSH-Px) assay kit, and catalase (CAT) assay kit were obtained from Nanjing Jiancheng Bioengineering Institute (Nanjing, China).

### 2.2. BOO Model and Cystometry Preparation

18 male Sprague-Dawley (SD, 8 weeks old) rats weighing 180 to 200 g were obtained from the Animal Center of Ninth People's Hospital of Shanghai Jiao Tong University School of Medicine. All rats were housed three per cage in a room under a 12 h light/dark cycle with free access to food and tap water under the condition of 20–26°C with 40–60% relative humidity. All experimental procedures were approved by the Ethics Committee of Shanghai Jiao Tong University School of Medicine.

The SD rats were randomly divided into three groups: the sham-operated group (*n* = 6), the BOO group (*n* = 6), and the BOO+SFN group (*n* = 6). The sham-operated group that underwent a sham operation was used as the control group, and BOO surgeries were applied to rats of the BOO group and the BOO+SFN group according to the method described previously [14]. Briefly, the rats were anesthetized with 10% chloral hydrate (3 mL/kg, intraperitoneally) and then fixed in a supine position. After sterilization with the iodine cotton ball in the abdomen area, abdominal midline incision (about 1.0 cm) was made and the bladder and proximal urethra were exposed. A 19-G needle was placed around the proximal urethra, and proximal urethra was loosely tied with the needle using 3-0 silk thread. The needle was then removed and incision was closed. Gentamycin (1 mL/kg) was given intramuscularly after surgery. Sham operations were performed in an identical manner without tying the silk thread. From the first day after operation, rats of the BOO+SFN group received daily intraperitoneally injections of SFN (0.5 mg/kg). Since SFN was dissolved in ethanol and then diluted with PBS, rats of the sham group and BOO group were given the same volume of PBS containing ethanol. The following experiments were conducted four weeks postoperation.

For the cystometric analysis in the rats of all groups, a catheter was placed in the bladder three days before cystometric analysis, as previously described [15, 16]. Briefly, flaring the end of the polyethylene tubing 50 (PE-50) to become a balloon serves as an anchor to maintain the tube within the bladder. Rats were anesthetized as above; a 1 cm incision was made on the dorsum between the scapulas. Then we developed a plane between the skin and the underlying muscle to create a tunnel around the ventral abdomen. After an abdominal midline incision was made, we exposed the bladder, grasped the smooth end of the PE-50 tubing with the clamp, and pull it back through the dorsum incision. The bulbed end was placed in the bladder dome and then the purse string suture around the tubing was pulled tight. The dorsal and abdominal incisions were closed and the animals were subjected to cystometric analysis subsequently.

### 2.3. Cystometric Analysis

The conscious rats were placed in a metabolic cage. For cystometry, the indwelling tubing was attached to a two-way valve that was connected to a pressure transducer as well as an infusion pump. We purged the system of any air bubbles and ensured continuous flow from the infusion pump. We infused saline into the bladder at a rate of 12 mL/h at room temperature in all groups. The cystometric parameters such as maximal pressure, bladder capacity, and others were measured. The rats were euthanized after the experiment and the bladder was collected for further study.

### 2.4. Histological Examination and Immunohistochemical Staining

Bladders were fixed in 4% paraformaldehyde, embedded in paraffin, and sectioned at 5 *μ*m. Bladder sections were processed for hematoxylin and eosin (HE) staining to observe general morphology. Masson trichrome staining was performed to evaluate the level of tissue fibrosis. Immunohistochemical staining was conducted as previously described. Briefly, the sections were subjected to heat for antigen retrieval with 10 mM sodium citrate buffer (pH 6.0). The primary antibodies, secondary antibodies, and DAB detection kit were used according to the manufacturer's instructions. Histological analysis was performed by two pathologists in a blinded manner.

### 2.5. TUNEL Assay

The one-step TUNEL apoptosis assay kit was used to measure the apoptosis level in the bladder smooth muscle according to the manufacturer's instructions. Briefly, the sections were regularly hydrated and immersed in 1% Triton X-100; then, the sections were incubated with Proteinase K solution for 30 minutes. The sections were reacted with TdT solution for 1 hour and Streptavidin-TRITC solution for 30 minutes, respectively, in a humidified and dark chamber. Finally, the sections were stained with DAPI for 10 minutes to stain cell nuclei. The apoptotic cells were observed under a fluorescence microscope. The percentage of apoptotic cells was measured.

### 2.6. Oxidative Stress Marker Determination

The oxidative stress marker was conducted as previously described [17, 18]. The content of MDA, GSH-Px, total SOD, and CAT in the bladder tissues was measured by spectrophotometry according to the manufacturer's protocols. Briefly, the MDA level was detected using the thiobarbituric acid (TAB) method and the maximum absorbance was at 532 nm. The measurement of total SOD activity was based on the combination of xanthine and xanthine oxidase, and the absorbance was read at 550 nm. The GSH-Px activity was measured using the enzyme-catalyzed reaction product (reduced glutathione) and the absorbance was recorded at 412 nm. The CAT activity was measured by the reaction of hydrogen peroxide with ammonium molybdate, and the absorbance measurement was done at 405 nm. Moreover, the MDA level in serum was also measured.

### 2.7. Protein Extraction and Western Blotting

Three rats of each group were randomly selected for western blotting. Total protein was extracted from frozen bladder tissues by trypan-blue in RIPA buffer containing protease inhibitors. Muscles lysates were centrifuged at 10000 g/min at 4°C for 10 min, and the supernatant was collected and used as total protein extracts. The nuclear protein was extracted according to the manufacturer's instructions of nuclear protein extraction kit. Briefly, bladder tissues were cut in pieces and homogenized in cytoplasm protein extraction reagent containing protease inhibitors, the lysates were centrifuged at 10000 g/min at 4°C for 5 min, the precipitation was collected and mixed with nucleoprotein extraction reagent for 30 min on ice, the lysates were centrifuged at 10000 g/min at 4°C for 10 min, and the supernatant was collected and used as nuclear protein extracts. Protein concentrations were measured by BCA protein assay. The samples were run on 10% SDS-polyacrylamide gels (20 *μ*g/lane), and then, proteins were transferred to PVDF membranes by electroblotting (200 mA). PVDF membranes were incubated in 5% BSA for 2 hours at room temperature, followed by three 5-minute washes in TBST. The PVDF membranes were then incubated overnight at 4°C with anti-Nrf2 (1 : 1000), HO-1 (1 : 1000), NQO1 (1 : 1000), bcl-2 (1 : 2000), Bax (1 : 2000), and GAPDH (1 : 2000) antibodies. GAPDH was used as internal normalizer. Then the membranes were washed three times for 10 min in TBST and incubated with goat anti-rabbit conjugated to horseradish peroxidase (1 : 2000) antibody for 2 hours and finally examined by chemiluminescence.

### 2.8. Statistical Analysis

All data are shown as mean ± SD. SPSS 16.0 was used to evaluate data. Differences between groups were analyzed using one-way ANOVA with *P* < 0.05 considered significant.

## 3. Results

### 3.1. Effect of SFN on Body and Bladder Weight

Body weight of rats was significantly lower in the BOO group than in the sham-operated group and BOO+SFN groups. The difference was caused by a slower weight increase instead of weight loss. The bladder weight in the BOO group was higher than that in the sham-operated group. And the bladder weight in the BOO+SFN group was higher than that in the BOO group (Table 1). No other side effects were found.

### 3.2. Improvement of Cystometric Outcomes in Partially Bladder Obstruction of Rats

**Figure 1:**
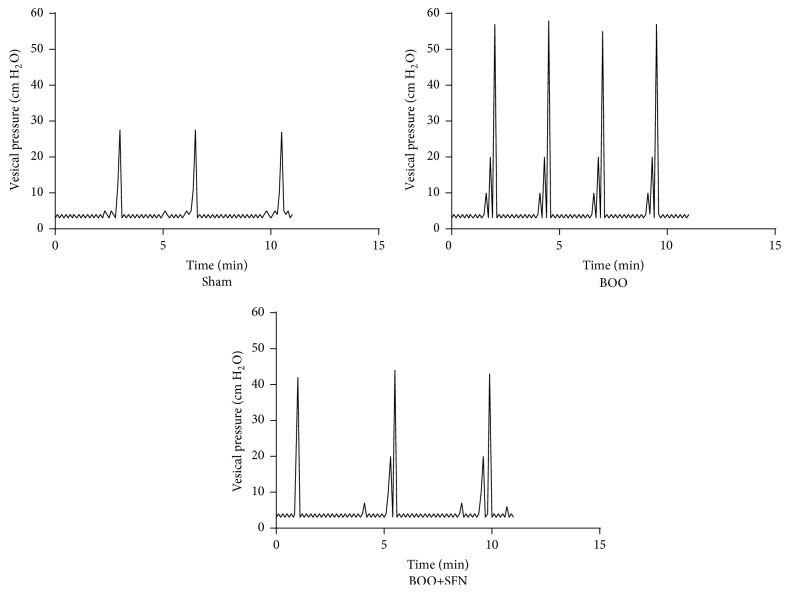
Effect of SFN on urodynamic changes in conscious BOO rats.

The parameters of cystometry were measured according to the urodynamic curve (Figure 1). The bladder pressure was significantly increased in obstructed rats relative to sham rats. However, the peak voiding pressure of bladders in obstructed rats treated by SFN is lower than obstructed rats at the 4-week time point. Bladder capacity was significantly higher in BOO rats compared to sham rats. Rats of the BOO+SFN group showed the highest bladder capacity among all groups. Bladder compliance decreased significantly at the 4-week time point after BOO. However, SFN treatment rescued the compliance deterioration possibly via a massive increase in bladder capacity. Moreover, we found that the interval of micturition was shorter in BOO rats than in sham rats. The micturition interval in BOO+SFN rats was significantly increased compared to BOO rats (Table 2).

### 3.3. Effect of SFN on BOO Induced Histological Changes in Bladder Detrusor

**Figure 2:**
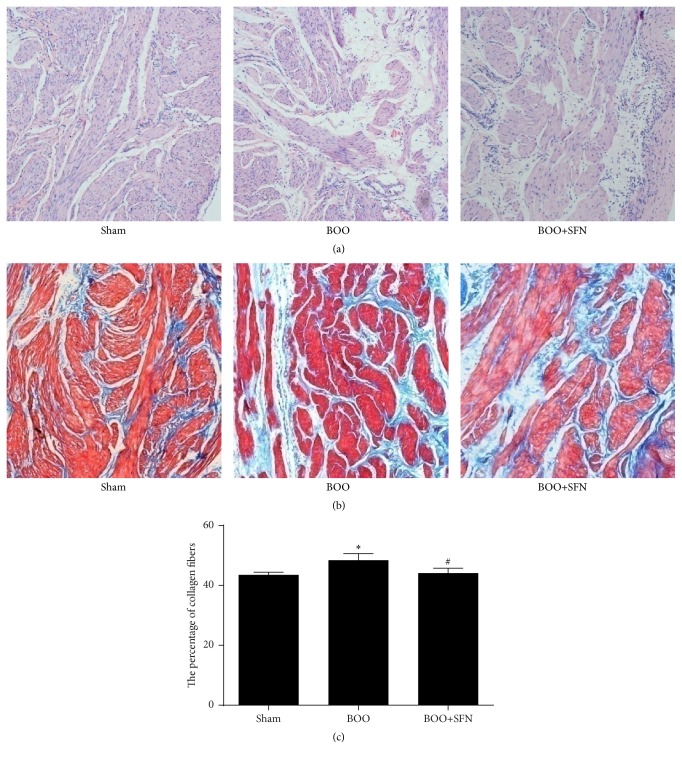
Effect of SFN on bladder histological changes in BOO rats. Original magnification ×100. (a) HE staining in sham, BOO, and BOO+SFN bladders; (b) Masson trichrome staining in sham, BOO, and BOO+SFN bladders; (c) the percentage of collagen fibers in muscular layer in sham, BOO, and BOO+SFN bladders, ^*∗*^ *n* = 6, *P* < 0.05 versus sham group, ^#^ *n* = 6, *P* < 0.05 versus BOO group.

HE staining showed that SFN had some protective effect on BOO bladder (Figure 2(a)). BOO caused obvious histological changes, such as the structural damage of detrusor smooth muscle. However, treatment with SFN significantly alleviated these histological changes in the bladders of BOO rats. The area ratio of collagen fibers was 43.55 ± 0.86, 48.40 ± 2.25, and 44.13 ± 1.64 in sham group, BOO group, and BOO+SFN group, respectively. An increase of collagen fibers in the muscular layer was observed in the BOO group and this increase was suppressed in the BOO+SFN group (Figures 2(b) and 2(c)).

### 3.4. Effect of SFN on Attenuating Oxidative Stress in BOO Rats

**Figure 3:**
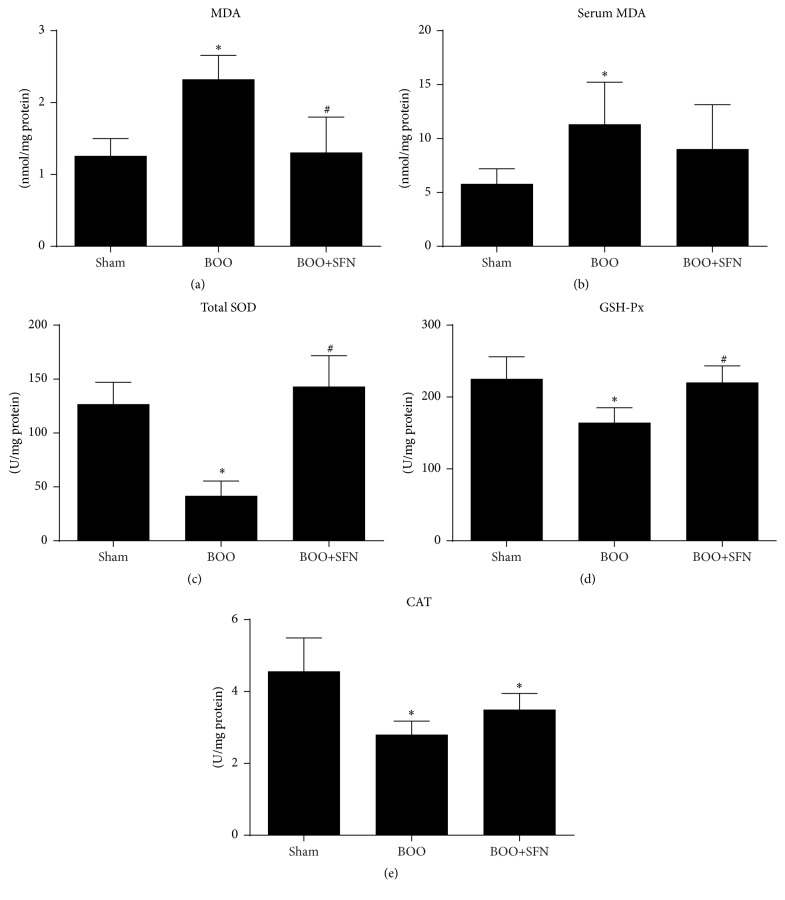
Effects of SFN on attenuating oxidative stress in BOO rats. (a) MDA level in the bladder of the three groups, ^*∗*^ *n* = 6, *P* < 0.05 versus sham group. ^#^ *n* = 6, *P* < 0.05 versus BOO group. (b) MDA level in serum of the three groups, ^*∗*^ *n* = 5, *P* < 0.05 versus sham group. (c) The activity of total SOD in the bladder of the three groups, ^*∗*^ *n* = 6, *P* < 0.05 versus sham group. ^#^ *n* = 6, *P* < 0.05 versus BOO group. (d) The activity of GSH-Px in the bladder of the three groups, ^*∗*^ *n* = 6, *P* < 0.05 versus sham group. ^#^ *n* = 6, *P* < 0.05 versus BOO group. (e) The activity of CAT in the bladder of the three groups, ^*∗*^ *n* = 6, *P* < 0.05 versus sham group.

The level of MDA, total SOD, GSH-Px, and CAT were measured to evaluate the level of oxidative stress. Our results showed that the content of MDA in bladder was significantly increased in BOO rats compared to sham rats (2.32 ± 0.33 versus 1.26 ± 0.24). However, the level of MDA was decreased in BOO+SFN rats (1.31 ± 0.49) (Figure 3(a)). The activities of total SOD, GSH-Px, and CAT were significantly decreased in BOO rats compared to sham rats (41.75 ± 13.59 versus 126.73 ± 20.31; 164.46 ± 20.64 versus 225.42 ± 30.65; 2.80 ± 0.37 versus 4.56 ± 0.93), respectively. However, treatment with SFN in BOO rats could significantly increase the total SOD, GSH-Px, and CAT activities (Figures 3(c), 3(d), and 3(e)). The MDA level in serum was also significantly elevated in BOO rats (Figure 3(b)).

### 3.5. Effect of SFN on Cell Apoptosis and Proliferation in the Bladder of BOO Rats

**Figure 4:**
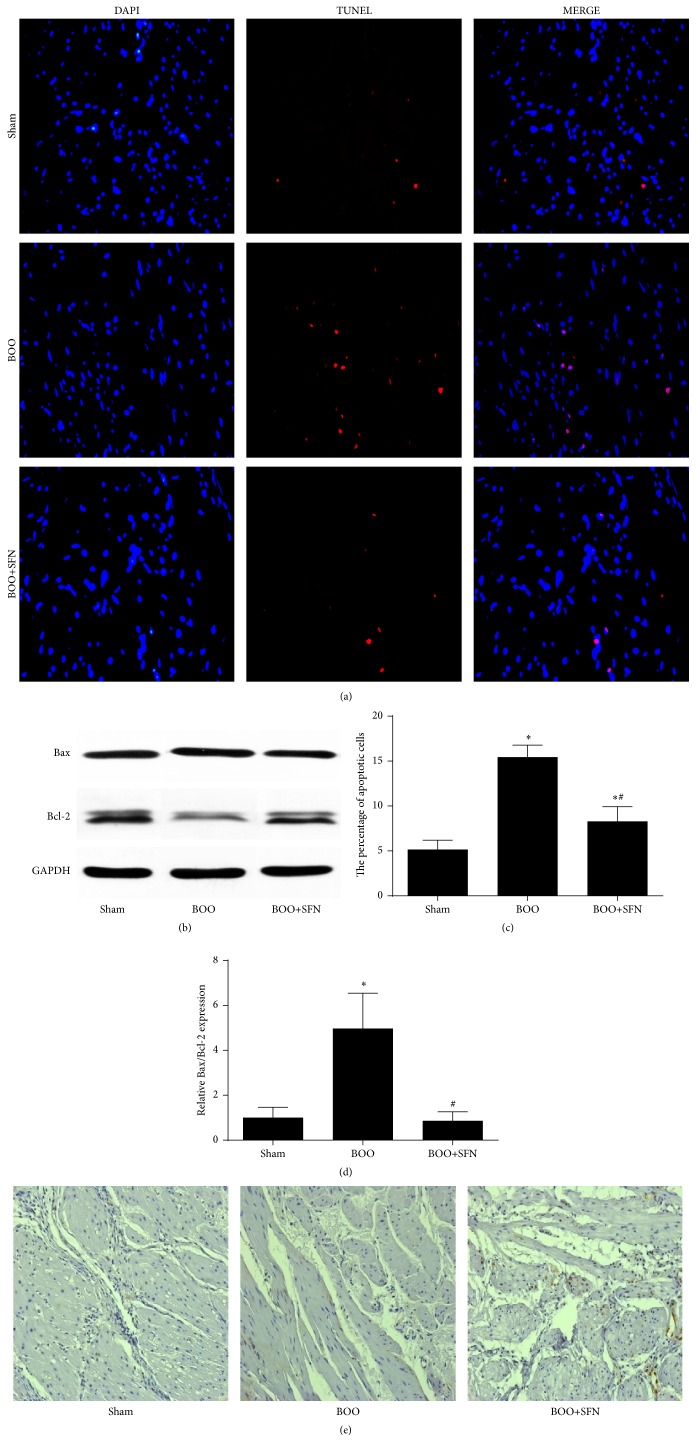
Effect of SFN on cell apoptosis and proliferation in BOO rats. (a) TUNEL staining showing the cell apoptosis level of the bladder in the three groups. Original magnification ×400. (b) The protein expression of Bax/Bcl2 ratio in the bladder of the three groups. (c) The statistical results of TUNEL staining in the three groups. ^*∗*^ *n* = 6, *P* < 0.001 versus sham group. ^#^ *n* = 6, *P* < 0.001 versus BOO group. (d) The statistical results of protein expression of Bax/Bcl2 ratio in the bladder of the three groups. ^*∗*^ *P* < 0.05 versus sham group. ^#^ *P* < 0.05 versus BOO group. (e) The expression of PCNA by immunohistochemical staining in the three groups. Original magnification ×200.

TUNEL staining was measured to observe whether SFN had protective effects on the cell apoptosis level of bladder (Figures 4(a) and 4(c)). The number of apoptotic cells in the bladder of BOO rats was markedly increased compared to sham rats (*P* < 0.001), whereas SFN treatment decreased the number of apoptotic cell in the bladder of BOO rats (*P* = 0.001). In accordance with the result of TUNEL staining, western blotting showed that the Bax/Bcl2 expression ratio was significantly increased in the bladder of BOO rats; however, this ratio was decreased in BOO+SFN rats (Figures 4(b) and 4(d)). Moreover, the immunohistochemical staining of PCNA showed that SFN did not cause obvious increase on cell proliferation in BOO+SFN group compared to BOO group (Figure 4(e)).

### 3.6. Effects of SFN on Bladder via Activation of the Nrf2-ARE Pathway

**Figure 5:**
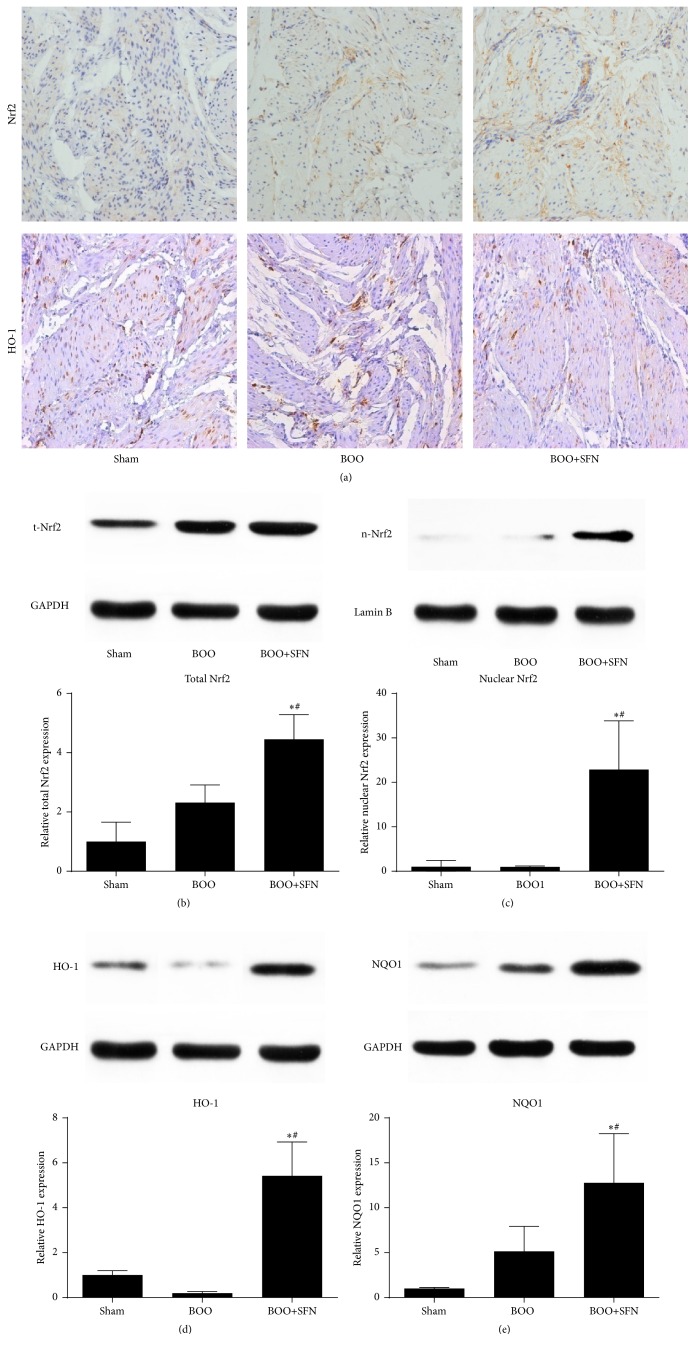
Effect of SFN on the Nrf2-ARE pathway in BOO rats. (a) Immunohistochemical staining of Nrf2 and HO-1 in the bladder of the three groups. Original magnification ×200. (b) The protein expression of total Nrf2 in the bladder of the three groups. (c) The protein expression of nuclear Nrf2 in the bladder of the three groups. (d) The protein expression of HO-1 in the bladder of the three groups. (e) The protein expression of NQO1 in the bladder of the three groups. ^*∗*^ *P* < 0.05 versus sham group. ^#^ *P* < 0.05 versus BOO group.

The transcription factor Nrf2 is a vital mediator involved in regulating cellular antioxidative responses. As SFN might be an activator of Nrf2, we investigated the effects of SFN on the expression of Nrf2 and its downstream target proteins. As shown in Figure 5(a), immunohistochemical results demonstrated that the expression level of Nrf2 was significantly higher in the muscular layers of the bladder in the BOO+SFN group compared to the BOO group. More importantly, the expression of Nrf2 was mainly located in the nucleus of the bladder cells. The expression of Nrf2 in the cell and nucleus was also measured by western blotting (Figures 5(b) and 5(c)). The results showed that the expression of Nrf2 was significantly increased in the bladder of BOO+SFN group compared with the BOO group and the total Nrf2 was increased in the bladder of the BOO group compared to the sham group. However, the expression of Nrf2 in the nucleus showed no significant difference between the BOO group and the sham group, and the level of Nrf2 in the nucleus was markedly increased in the BOO+SFN group compared to the BOO group.

We then studied the antioxidative function of Nrf2 by measuring the expression of its downstream target proteins, HO-1 and NOQ1. As shown in Figures 5(a) and 5(d), the expression of HO-1 was increased in the BOO+SFN group compared to the BOO group, which was consistent with the result of Nrf2 expression in the nucleus. However, we found that the level of NQO1 in the BOO group was higher compared to the sham group,but lower than the BOO+SFN group (Figure 5(e)).

## 4. Discussion

Evidences suggest that oxidative stress plays a critical role in bladder outlet obstruction-mediated bladder dysfunction [19]. Therefore, the development of new means to reduce oxidative stress through inducing the endogenous phase 2 enzymes can be considerably attractive. The use of an appropriate animal model is of great significance for understanding the factors responsible for the pathophysiological changes of a disease. As our previous research proposed, PBOO-induced bladder remodeling in a rat model is similar to that in patients with BPH. In the present study, we found that our rat model was a useful model to study structural and functional alterations in the bladder.

In this study, we found that the weight of bladder was significantly higher in the BOO rats when treated with SFN compared to BOO rats. In order to reflect the real situation more accurately, we performed urodynamic studies in conscious rats. The cystometric parameters such as maximal pressure, bladder capacity, compliance, and micturition interval were measured as reported [20]. We found that the capacity, micturition interval, and compliance were significantly increased and the peak voiding pressure had some extent of reduction by treatment with SFN in BOO rats. As an activator of Nrf2, SFN has been shown to activate the Nrf2 expression and its target antioxidant genes [21, 22]. The daily dose of 0.5 mg/kg SFN in this study has been proved effective in other's research [23]. Moreover, in accord with a previous study [24], the histological staining showed that muscle bundles were severely damaged and collagen deposition was increased in BOO rats. This increase was suppressed after the 4-week treatment with SFN in BOO rats. These results indicate an inverse relationship between bladder compliance and collagen fibers and indicate that SFN could protect rats against BOO-induced dysfunction and morphological damage of the bladder.

In order to investigate whether SFN had antioxidant activity in the bladder of BOO rats, we measured MDA levels in the three groups. MDA level is used as an indicator of lipid peroxidation, as lipids in the cell membrane are destroyed to generate MDA in an amount proportional to the degree of tissue destruction. Our results showed that BOO significantly increased the level of MDA in the bladder and serum. However, the increase in the level of MDA was partially suppressed in SFN treated BOO rats, which was consistent with studies of other antioxidants, such as phytotherapeutic agents [6]. The activities of redox status markers (total SOD, GSH-Px, and CAT) were also measured. We found that the activities of these markers were significantly decreased in the bladder of the BOO group compared to the sham group; however, SFN significantly enhanced their activities in BOO rats, which is consistent with other literature [18]. These results suggested that SFN could alleviate oxidative stress by increasing the activities of antioxidative enzymes in BOO rats.

Cell apoptosis is an important factor that leads to bladder dysfunction in BOO model. In our previous study, the number of apoptotic cells was significantly increased in the BOO group compared to the sham group. Activation of the Nrf2-ARE signaling pathway was reported to protect various tissues against apoptosis [25, 26]. TUNEL staining results showed that the treatment of SFN in BOO rats could significantly decrease the number of apoptotic cells; in accordance with this, we found that the expression of the Bax/Bcl2 ratio in the bladder was also decreased in BOO rats when treated with SFN. These results suggested that SFN might protect bladder against BOO-induced apoptosis via decreasing the expression of Bax/Bcl2 ratio. Meanwhile, we also assessed the effect of SFN on cell proliferation by PCNA staining. The result showed that the level of cell proliferation was significantly higher in the bladder of BOO rats. However, SFN showed no obvious effect on cell proliferation in BOO+SFN group compared to BOO group.

Many studies have shown that antioxidant could ameliorate bladder dysfunction in BOO rats [6, 16]. However, the exact mechanism has not been clarified. The Nrf2-ARE signaling pathway is known to exert antioxidative effects on various diseases. SFN treatment can protect various tissues against oxidative stress via the Nrf2-ARE signaling pathway [10, 22]. Whether SFN could ameliorate bladder dysfunction in BOO model has not yet been reported. In the present study, we first reported the effect of SFN on the bladder of BOO rats and changes of the Nrf2-ARE signaling pathway. By immunohistochemical staining and western blot (Figures 5(a) and 5(b)), we found that the expression of Nrf2 protein in the bladder of BOO rats with SFN treatment was significantly increased compared to BOO rats. In addition, the Nrf2 expression in the nucleus was not significantly changed between the BOO rats and the sham rats. However, the level of Nrf2 in the nucleus was markedly increased in the BOO rats when treated with SFN (Figure 5(c)). The result indicated that SFN could promote the transportation of Nrf2 into nucleus and the transcription of its target antioxidant genes. We next measured the expression level of HO-1 and NQO1, the downstream genes of Nrf2-ARE pathway by western blot. The levels of HO-1 and NQO1 increased significantly in BOO rats after SFN treatment. In addition, the changes of total SOD, GSH-Px, and CAT activity were consistent with the Nrf2 expression in the nucleus (Figures 3(c), 3(d), and 3(e)). Above all, our results suggested that SFN could promote Nrf2's translocation into the nucleus and the expression of its target antioxidant genes, which ameliorated the oxidative stress in BOO rats.

Up to now, there is no standard protocol of when to perform cystometry postoperation. In this study, we found that an interval of 3 days is necessary for rats to recover from the surgery, which was consistent with other's report [16]. In addition, we did not apply SFN gradients in the treatment, as we found that the dose of 0.5 mg/kg had significantly protective effects on BOO rats in our preexperiments. Although SFN is extracted from natural plants, the side effects should be investigated in the future.

In conclusion, our study is the first to demonstrate that SFN has significant protective effects against oxidative stress and could ameliorate bladder dysfunction through activation of the Nrf2-ARE pathway in the bladder of BOO rats. SFN can alleviate pBOO-induced bladder injury via activating Nrf2 pathway and suppressing cellular apoptosis. Although there is insufficient evidence to extrapolate results from fundamental research to humans, our study could support the potential application value of SFN as treatment of bladder dysfunction associated with BPH and LUTS. These findings indicated that Nrf2 activator might be recommended to protect against bladder dysfunction in BOO related diseases.

## Figures and Tables

**Table 1:** Body and bladder weight before operation and at sacrifice.

	Sham	BOO	BOO+SFN
Body weight (g):			
Preop	171.67 ± 5.16	175 ± 7.07	175 ± 8.94
4-week BOO	321.67 ± 8.16	275 ± 14.14^*∗*^	306.67 ± 21.6^#^
Bladder weight (mg):			
4-week BOO	125.67 ± 15.97	314.5 ± 78.03^*∗*^	616.17 ± 250.34^*∗*#^

^*∗*^Significantly different versus sham group (*P* < 0.05).

^#^Significantly different versus BOO group (*P* < 0.05).

**Table 2:** Outcomes of cystometric parameters in conscious rats.

	Sham	BOO	BOO+SFN
Peak voiding pressure (cm H_2_O)	27.60 ± 3.51	50.4 ± 7.44^*∗*^	42.38 ± 5.6^*∗*#^
Capacity (mL)	1.05 ± 0.27	3.83 ± 0.68^*∗*^	5.42 ± 0.58^*∗*#^
Compliance (*μ*L/cm H_2_O)	26.61 ± 2.73	11.37 ± 1.76^*∗*^	18.52 ± 3.21^*∗*#^
Micturition interval (min)	3.47 ± 0.48	2.24 ± 0.38^*∗*^	4.49 ± 0.74^*∗*#^

^*∗*^Significantly different versus sham group (*P* < 0.05).

^#^Significantly different versus BOO group (*P* < 0.05).

## Abbreviations

ARE: Antioxidant response element
BOO: Bladder outlet obstruction
BPH: Benign Prostatic Hyperplasia
CAT: Catalase
GSH-Px: Glutathione peroxidase
HE: Hematoxylin and eosin
HO-1: Heme oxygenase-1
Keap1: Kelch-like ECH-associated protein 1
LUTS: Lower urinary tract symptoms
MDA: Malondialdehyde
NQO1: NAD(P)H: Quinone oxidoreductase 1
Nrf2: Nuclear erythroid related factor 2
PE-50: Polyethylene tubing 50
ROS: Reactive oxygen species
SD: Sprague-Dawley
SFN: Sulforaphane
SOD: Superoxide dismutase
TUNEL: TdT-mediated dUTP Nick-End Labeling.
